# Breakfast skipping and cardiometabolic risk factors in adolescents: Systematic review

**DOI:** 10.11606/s1518-8787.2021055003077

**Published:** 2021-12-01

**Authors:** Marielly Rodrigues Souza, Morgana Egle Alves Neves, Bartira Mendes Gorgulho, Amanda Moura Souza, Patrícia Simone Nogueira, Márcia Gonçalves Ferreira, Paulo Rogério Melo Rodrigues

**Affiliations:** I Universidade Federal de Mato Grosso Faculdade de Nutrição Programa de Pós-Graduação em Nutrição, Alimentos e Metabolismo Cuiabá MT Brasil Universidade Federal de Mato Grosso. Faculdade de Nutrição. Programa de Pós-Graduação em Nutrição, Alimentos e Metabolismo. Cuiabá, MT, Brasil; II Universidade Federal de Mato Grosso Faculdade de Nutrição Departamento de Alimentos e Nutrição Cuiabá MT Brasil Universidade Federal de Mato Grosso. Faculdade de Nutrição. Departamento de Alimentos e Nutrição. Cuiabá, MT, Brasil; III Universidade Federal do Rio de Janeiro Instituto de Estudos em Saúde Coletiva Departamento de Epidemiologia e Bioestatística Rio de Janeiro RJ Brasil Universidade Federal do Rio de Janeiro. Instituto de Estudos em Saúde Coletiva. Departamento de Epidemiologia e Bioestatística. Rio de Janeiro, RJ, Brasil

**Keywords:** Adolescent, Breakfast, Food Deprivation, Metabolic Syndrome, Cardiometabolic Risk Factors, Review

## Abstract

**OBJECTIVE:**

To systematically review the results of the association between breakfast skipping and cardiometabolic risk factors in adolescents.

**METHODS:**

The articles were searched in May 2020 from PubMed, Virtual Health Library, Scopus, Web of Science and Scientific Electronic Library Online (SciELO). The review included observational studies conducted with adolescents (10–19 years old), which estimated the association of breakfast skipping with at least one outcome (markers of body adiposity, blood pressure, serum lipid and glucose levels). Regarding the risk of bias, the articles were evaluated using the Research Triangle Institute (RTI) Item Bank on bias risk and accuracy of observational studies. The quality of the evidence was assessed by the Grade rating.

**RESULTS:**

A total of 43 articles involving 192,262 participants met the inclusion criteria and were considered in this review. The prevalence of breakfast skipping ranged from 0.7% to 94% and 60.5% of studies were classified with low risk of bias. The significant association between breakfast skipping and cardiometabolic risk factors was found in twenty-nine cross-sectional articles (n = 106,031) and four longitudinal articles (n = 5,162) for excess adiposity, in three articles (n = 8,511) for high total cholesterol levels, low-density lipoprotein and triglycerides, and in three studies (n = 6,303) for high blood pressure levels. However, there was no significant association between breakfast skipping and glycemic profile. According to the Grade rating, all the associations had low quality of evidence.

**CONCLUSION:**

The results of this review suggest that breakfast skipping is associated with cardiometabolic risk factors in adolescents aged 10 to 19 years. However, considering the low quality of the evidence, the present results should be interpreted carefully. In addition, our findings highlight the importance of standardizing the definition of breakfast skipping and that more prospective studies are needed to determine how skipping breakfast can affect cardiometabolic risk factors in the long time.

## INTRODUCTION

In recent decades, the meal consumption pattern has been associated with adolescents’ physical growth and various health outcomes^1^. Among behaviors related to that, we highlight the unfavorable effect of skipping meals, especially breakfast^1^.

Breakfast is the first and most important meal^2^ of the day, and contributes to maintaining adolescents’ overall health and diet quality^3^. This meal usually occurs before the start of daily activities, after a period of rest (sleep) of approximately 8 to 10 hours without any food consumption^4^.

Regular consumption of breakfast has been considered as a marker of healthy eating habits^3,5^, and plays an important role in nutritional adequacy, bone and cardiovascular health^6,7^, school performance, cognitive performance^8^, and improved attention and mood^9^.

In adolescence, unhealthy eating habits, such as skipping breakfast, can compromise growth, development, and long-term health^10^. Skipping this meal is also associated with cardiometabolic risk factors in adolescents, such as increased central and total adiposity, insulin resistance, and dyslipidemia^5,10^.

The term cardiometabolic risk was proposed by the American Diabetes Association and the American Heart Association, and it is used to describe a set of clinical signs, such as: dyslipidemia, insulin resistance, obesity, and high blood pressure^13,14^.

Some physiological and metabolic mechanisms may explain how breakfast skipping may be associated with cardiometabolic risk factors, including its contribution to an unfavorable energy balance causing weight gain and changes in serum lipid levels^15^. Another explanation is that breakfast skipping increases hunger and leads to excessive consumption throughout the day, resulting in increased body weight^16^. Prolonged fasting time alters glucose homeostasis, resulting in decreased insulin secretion and intact glucagon-like peptide-1 (iGLP-1) responses and increased post meal plasma glucose^17^.

Therefore, this study sought to systematically review the association between breakfast skipping and cardiometabolic risk factors in adolescents.

## METHODS

This is a systematic review of the literature, aiming to answer the questions: “Is breakfast skipping associated with serum lipid and glucose levels in adolescents?”; “Is breakfast skipping associated with blood pressure in adolescents?”; “Is breakfast skipping associated with markers of body adiposity in adolescents?” This review was developed and structured according to the acronym PECO components (Population: adolescents; Exposure: breakfast skipping; Control: without control; Outcome: Lipid and glycemic profile, high blood pressure, systemic arterial hypertension, and markers of body adiposity). The protocol was registered with the International Prospective Register of Systematic Reviews (PROSPERO), National Institute for Health Research, under the registration number: CRD42018105003.

The records were searched independently by two researchers in the following databases: MEDLINE via PubMed, Web of Science (WoS), Scopus, Virtual Health Library (VHL) and SciELO. The searches were carried out in May 2020. No country restrictions have been established. Descriptors were used for skipping breakfast, blood pressure, lipid and glycemic profile, and adolescents, combining the terms through the Boolean operators OR and AND, as shown in Supplementary Table 1.

**Table 1:** General characteristics of the studies included in the systematic review, main results, and scores in the risk of bias assessment.

Author/Place N/Age/Sex	Breakfast skipping assessment	Confounding factors	Prevalence of breakfast kipping	Main results				Risk of bias Points^1^

				Markers of body adiposity	Lipid profile	Glycemic profile	Blood pressure	

Cohort studies								

Timlin et al.^21^ Minnesota n = 2,216 Age mean: time 1 = 14.9 ± 1.6 years, time 2 = 19.4 ± 1.7 years M/F^2^ Period^3^: 5 years	“During the last week, how many days did you have breakfast?” The frequency of breakfast was recoded into three categories: Daily (every day) Intermittent (1 to 6 days/week) Never (daily omitters)	Age, sex, race, NSE, exercise, cigarettes, alcoholic beverage, total energy, carbohydrates, fiber, food items (milk, cold cereals, juices and bread), psychosocial variables, fast eating, skipping weight management meals, dieting last year, ate little last year for weight loss, be teased about weight, concern about the current weight.	Time 1: 16.4% (girls) and 13.0% (boys). Time 2: 13.8% (girls) and 18.9% (boys).	Adolescents who never ate breakfast had a higher mean increase in BMI (model 1: 2.2 ± 0.19, model 2: 2.2 ± 0.20, p < 0.05) than those who consumed daily (model 1: 1.6 ± 0.16, p < 0.05) and intermittent (model 1: 2.2 ± 0.09, p < 0.05).	Outcome not evaluated.	Outcome not evaluated.	Outcome not evaluated.	9 (Present).
Wang et al.^16^ New Haven n = 1,534 F 55.0% 4^th^, 5^th^, 6^th^ grade Period^3^: 4 years	(1) Frequent skippers (2) Inconsistent school eaters (3) Inconsistent home eaters (4) Regular home eaters (5) Regular school eaters (6) Double breakfast eaters.	Schooling, year of follow-up, weight status, classroom breakfast program, race/ethnicity.	It has progressively increased over time (5^th^ grade: 11.5%; 6^th^ grade: 17.5%; 7^th^ grade: 22.9%).	Adolescents in the group that frequently skipping breakfast were more likely to be overweight and obese compared to adolescents in the group that consumed double breakfast (OR = 2.66, 95%CI = 1.67; 4.24).	Outcome not evaluated.	Outcome not evaluated.	Outcome not evaluated.	10 (Low).
Cayres et al.^22^ Brazil n = 86 11–14 years F 51.2% Period^3^: 12 months.	“How many days a week do you usually have breakfast? Zero (score 0)^a^. 1 to 2 days (score 1)^a^ 3 to 5 days (score 2)^a^ Every day of the week (score 3).	Sex, age and level of sexual maturation.	Not shown.	At the beginning of the study, adolescents who skipping breakfast had higher BMI (p = 0.032), abdominal fat (p = 0.019) and total body fat (p = 0.012) than those who ate breakfast regularly. After 12 months of follow-up abdominal fat (0.4% [95%CI = −1.2; 2.2]) and total body fat (0.08% [95%CI = −0.8; 1.0]) of adolescents who skipped breakfast increased more than those who ate regularly.	Outcome not evaluated.	Outcome not evaluated.	Outcome not evaluated.	11 (Low).
Hassan et al.^23^ Brazil n = 809 10–16 years M 53,8% Period^3^: 3 years.	Breakfast Frequency: Never or almost never^a^ 1 to 2 times a week 3 to 4 times a week 5 to 6 times a week Daily	Weight status, family breakfast and diet.	9.6%	There was no association between skipping breakfast and weight status in girls (RR = 0.88, 95%CI = 0.53; 1.45) and boys (RR = 1.29; 95%CI = 0.73; 2.30).	Outcome not evaluated.	Outcome not evaluated.	Outcome not evaluated.	11 (Low).
Hassan et al.^24^ Brazil n = 809 10–16 years M/F^2^ Period^3^: 3 years.	Breakfast consumption frequency: Never or almost never (no consumption) * 1 to 4 times/week (Intermediate Frequency) 5 times/week (Regular Frequency).	Type of school (public and private), screen time, diet, sexual maturation and energy expenditure of physical activity.	9.6%	There was no significant association between skipping of breakfast, BMI and% BF over time (p > 0.05).	Outcome not evaluated.	Outcome not evaluated.	Outcome not evaluated.	10 (Low).
Wu et al.^11^ Taiwan n = 1,326; 10–18 years M 52,0% Period^3^: 5 years.	Skipping Breakfast Yes Not	Family structure, household income, and self-assessment of available money.	Not shown	Skipping breakfast was associated with overweight only in female adolescents (OR = 1.63, 95% CI = 1.20; 2.22).	Outcome not evaluated.	Outcome not evaluated.	Outcome not evaluated.	9 (Present).

Cross-Sectional studies								

Harding et al.^25^ London n = 6,599 11–13 years M 53.0%	Skipping breakfast sometimes or always vs. rarely.	NSE, family type, height, pubertal stage, age.	Not shown..	Skipping was associated with overweight (girls: OR = 1.66, 95%CI = 1.38; 2.01; boys OR = 1.53, 95%CI = 1.27; 1.84) and obesity (girls: OR = 1.74; 95%CI 1.30; 2.34; boys OR = 2.06; 95%CI = 1.57; 2.70)	Outcome not evaluated.	Outcome not evaluated.	Outcome not evaluated.	8 (Low).
Maddah^26^ Iran n = 2,090 14–17 years F 100%.	“How many times during the week do you have breakfast?” Never* 1 to 2 times/week Most times a week.	Maternal education, watching TV, walking, age, birth weight, and age at menarche.	Not shown.	Skipping breakfast was associated with overweight / obesity in both urban (OR = 1.96, 95%CI = 1.52; 2.35) and rural schools (OR = 2.23; 95%CI = 1.37; 3.65).	Outcome not evaluated.	Outcome not evaluated.	Outcome not evaluated.	9 (Low).
Mota et al.^27^ Portugal n = 886 13–17 years F 52.0%	Skipping breakfast: Yes^a^ Not	Not adjusted.	94.0% (boys) and 87.0% (girls)	Regardless of gender, breakfast skipping is not seen as a predictor of being at risk of obesity (Girls: OR = 0.98, 95%CI = 0.43; 2.20. Boys: OR = 1.39, 95%CI = 0.59; 3.31).	Outcome not evaluated.	Outcome not evaluated.	Outcome not evaluated.	7 (Present)
Sánchez et al.^28^ Gran Canaria n = 1,002 12–14 years M 50.0%	Breakfast: Yes Not*	Not adjusted.	8.0% (girls) and 4.4% (boys).	Adolescents who skipped breakfast had a higher prevalence of overweight and obesity than those who did not omit (girls: 30.0% vs 17.5%, p = 0.031; boys: 27.3 vs 18.2%, p = 0.028)	Outcome not evaluated.	Outcome not evaluated.	Outcome not evaluated.	7 (Present).
Alexander et al.^29^ California n = 110 10–17 years. M 59.1%	Breakfast was defined as consumption of any food or drink between 5:00 am and 10:00 am with combined total energy ≥ 100 kcal. R24h in 2 days (not eating breakfast on both days was defined as skipping). Breakfast Categorization: Breakfast eater; Occasional breakfast eater; Breakfast skipper.	Age, Tanner stage, sex, total fat, total lean tissue mass, and total energy consumed.	21.5%	There was no significant difference between breakfast consumption categories for BMI (p = 0.282), total lean mass (p = 0.796), total fat (p = 0.063) and subcutaneous abdominal fat (p = 0.817). The adolescents who skipped breakfast had higher intra-abdominal fat than occasional consumers (47.2 vs. 32.1 cm^2^, p = 0.004) and regular consumers (47.2 vs. 32.0 cm^2^, p = 0.05).	Outcome not evaluated.	There was no significant difference for acute insulin response (p = 0.212), insulin sensitivity (p = 0.077) and disposition index (p = 0.060).	Outcome not evaluated.	9 (Low).
Croezen et al.^30^ Netherlands n = 25,176 13–16 years F 51.2%	Skipping breakfast frequency: 7 days/week 5–6 days/week 3–4 days/week 1–2 days/week 0 days/week	Gender, family status, ethnicity, education and smoking.	Not shown.	Skipping breakfast 7 days/week was associated with overweight and obesity among adolescents 2^nd^ grade (OR = 2.17; 95%CI = 1.66; 2.85) and 4^th^ grade (OR = 1.75; 95%CI = 1.39; 2.21).	Outcome not evaluated.	Outcome not evaluated.	Outcome not evaluated.	8 (Low).
Kollias et al.^31^ Greece n = 558 12–17 years M 50.0%	Have breakfast: Yes Not^a^	Age and BMI.	68%	Outcome not evaluated.		Outcome not evaluated.	Skipping breakfast was associated with increased SBP in boys (2.81 ± 1.35 mmHg, p < 0.05).	9 (Low).
Maddah et al.^32^ Iran n = 2,255 14–18 years F 100%	“How many times during the week do you have breakfast?” Never 1–2 times/week 3 times or more per week.	Not adjusted.	Not shown.	Breakfast skipping was more prevalent in overweight/obese girls than in those with normal weight in urban (62.7 vs. 53.5%, p < 0.001) and rural (65.8 vs. 48.8; p < 0.001) areas.	Outcome not evaluated.	Outcome not evaluated.	Outcome not evaluated.	8 (Low).
Sun et al.^33^ Japan n = 5,753 12–13 years F 50.6%	Breakfast Consumption: Daily Almost daily Sometimes Rarely^a^	Age, paternal overweight, maternal overweight, and lifestyle variables.	1.1% (boys) and 0.7% (girls).	Breakfast skipping was associated with overweight (Boys: OR = 2.59, 95%CI = 1.05; 6.40; Girls: OR = 7.93, 95%CI = 2.79; 22.53).	Outcome not evaluated.	Outcome not evaluated.	Outcome not evaluated.	9 (Low).
Deshmukh-Taskar et al.^34^ United States of America n = 5,339 14–18 years M 51.6%	Consumption of any food or drink on a meal occasion named by the respondent as breakfast at R24h No consumption of food or drink, excluding water^a^.	Age, sex, ethnicity, NSE, physical activity, and energy intake.	31.5%	Those who skipped breakfast had higher WC (78.5 vs. 75.0 cm; p < 0.0167) and higher prevalence of obesity (20.7 vs. 13.2%, p < 0.0005) in comparison to consumers.	Outcome not evaluated.	Outcome not evaluated.	Outcome not evaluated.	9 (Low).
Kapantais et al.^35^ Greece n = 14,454 13–19 years F 53.8%	Do not eat anything for breakfast or have breakfast occasionally (< 2 times/week) – Breakfast skipped* Breakfast Consumption.	Not adjusted.	13.6% (boys) and 17.1% (girls).	Breakfast skippers had higher BMI than consumers (boys: 23.2 vs. 21.9 kg/m^2^, p < 0.001; girls: 21.9 vs. 20.9 kg/m^2^, p < 0.001).	Outcome not evaluated.	Outcome not evaluated.	Outcome not evaluated.	8 (Low).
Maddah and Nikooyeh ^36^ Iran n = 2,577 12–17 years F 100%	“How many times in a week do you have breakfast?” Rarely Most times a week Categorized in: Regular Not regular.	Age, birth weight, maternal education level, watching TV (hour per day), birth rate, maternal employment, place of residence, maternal overweight/obesity, paternal overweight/obesity, walking (hour / day).	Not shown.	Girls who skipped breakfast were more likely to be overweight/obese (OR = 1.4, 95%CI = 1.09; 1.93).	Outcome not evaluated.	Outcome not evaluated.	Outcome not evaluated.	9 (Low).
Thompson-McCormick et al.^37^ Fiji n = 517 15–20 years^3^; F 100%	“How many days in a week (on average) do you skip breakfast?” With response options ranging from 0 to 7 days.	Dimensions of Western/Ethnic and Fijian cultural orientation and involvement, age, school location, relative material wealth, boarding school, parental involvement, EDE-Q global score, and age.	68% skipping at least once/week and 41% skipping three or more times/week.	Adolescents who skipped breakfast were more likely to be overweight (OR = 1.15, 95%CI = 1.06; 1.26, p < 0.01).	Outcome not evaluated.	Outcome not evaluated.	Outcome not evaluated.	7 (Present).
Kim and So^38^ Korea n = 72,399 Age mean = 15.09 ± 1.75 M 52.7%	“Usually how many days a week did you have breakfast?” 1–2 day (s) 3–5 days 6 to 7 days No breakfast^a^.	Age, smoking, frequency of consumption, parental education, economic status, frequency of physical activity (vigorous and moderate), frequency of muscle strength exercises, mental stress, and sleep duration.	54.5%	There was no association between breakfast skipping and overweight (boys: OR = 1.059, 95%CI = 0.968; 1.159; girls: OR = 1.019, 95%CI = 0.992; 1.125) and obesity (boys: OR = 0.932, 95%CI = 0.854; 1.018; girls: OR = 0.941, 95%CI = 0.824; 1.073).	Outcome not evaluated.	Outcome not evaluated.	Outcome not evaluated.	8 (Low).
Kuriyan et al.^39^ Bangalore n = 3,737 10–16 years F 58.0%	Skip breakfast: Yes^a^ Not.	Not adjusted.	Not shown.	The adolescents who skipped breakfast had increased WC (+0.31 cm).	Outcome not evaluated.	Outcome not evaluated.	Outcome not evaluated.	7 (Present).
Vaezghasemi et al.^40^ Sweden n = 4,987 13–15 years M 50.5%	Breakfast on school days: ≥ 4 times/week ≤ 3 times/week^a^.	Age, country of birth, parental status, self-rated health, food consumption (fruits, vegetables, sweets, and snacks), tooth brushing, sleep duration, TV watching, physical activity, smoking, snuff use, alcohol use, and drug use.	Not shown.	Skipping breakfast was associated with overweight/obesity (boys: OR = 1.7, 95%CI = 1.4; 2.2; girls: OR = 1.6, 95%CI = 1.2; 2.1), after adjustment this association remained only for males (OR = 1.4, 95%CI = 1.1; 1.8, p = 0.016).	Outcome not evaluated.	Outcome not evaluated.	Outcome not evaluated.	5 (Present).
Shafiee et al.^41^ Iran n = 5,625 10–18 years M/F^2^	Breakfast Consumption: Regular consumption: 6-7 days/week; Often: 3 to 5 days/week; Seldom: 0–2 days/week^a^.	Age, sex, family history of chronic disease, mother’s education, parent’s education, physical activity, NSE, BMI in all abnormalities except for obesity.	29.0%	Those who rarely ate breakfast were more likely to have general obesity (OR = 1.47, 95%CI = 1.20; 1.82) and abdominal obesity (OR = 1.39, 95%CI = 1.04; 1.86).	Rarely consuming breakfast increased the chance of having high TG (OR = 1.41, 95%CI = 1.03; 1.93).	There was no association between fasting glucose and breakfast skipping (OR = 0.83, 95%CI = 0.64; 1.08).	The mean SBP was higher in the “rarely breakfast” group (p < 0.001).	8 (Low).
Boričic et al.^42^ Serbia n = 2,139 10–19 years M 50.4%	Breakfast Consumption: Every day Sometimes Never^a^.	Sex and Age.	Not shown.	Skipping breakfast was associated with being overweight (OR = 1.43; 95%CI = 1.02; 2.01).	Outcome not evaluated.	Outcome not evaluated.	Outcome not evaluated.	8 (Low).
Díez-Navarro et al.^43^ Madrid n = 986 11–15 years F 55.0%	Breakfast Presence No breakfast^a^.	Not adjusted.	7.5% of girls do not eat breakfast daily.	Between 11 and 13 years old: students who skipped breakfast had higher rates of obesity than those who did not omit (26.7% vs. 6.1%, p < 0.05).	Outcome not evaluated.	Outcome not evaluated.	Outcome not evaluated.	6 (Present).
Faizi et al.^44^ India n = 1,416 13–15 years M 50.3%	The frequency of breakfast was calculated based on two questions: Q1. In a normal week, how many times/week do you have your breakfast? Q2 Last week, how often did you have your breakfast? The final response was calculated as Q1 + Q2/2. Categorized in: < 2 times/week^a^ 3–5 times/week 6–7 times/week	Not adjusted.	6.2%	Skipping breakfast was associated with overweight/obesity (OR = 3.44, 95%CI = 2.08; 5.68). The mean BMI Z-score of breakfast-omitting adolescents (1.11) was higher than those who reported breakfast frequency 3–5 times/week (0.57) and 6–7. times/week (−0.42) with p = 0.001 in both cases.	Outcome not evaluated.	Outcome not evaluated.	Outcome not evaluated.	6 (Present).
Garcia-Continente et al.^45^ Barcelona n = 3,089 13–18 years F 52%	(1) Always have breakfast before leaving home and halfway morning (2) Always have breakfast before leaving home or in the middle of the morning. (3) Eat breakfast sometimes, but not every day before (4) Never eat breakfast^a^.	Age, Family Affluence Scale, ownership of the school, and NSE of the neighborhood of the school.	2.0%.	Skipping breakfast was associated with overweight only in females (OR = 4.07, 95%CI = 1.59; 10.44). Skipping breakfast was associated with obesity only for males (OR = 3.24; 95%CI = 1.16; 9.07).	Outcome not evaluated.	Outcome not evaluated.	Outcome not evaluated.	7 (Present).
Garg et al.^46^ India n = 195 10–16 years M 64.1%	Skipped breakfast Did not skip breakfast.	Not adjusted.	23.6%	There was no significant association between breakfast skipping and overweight (p = 0.992).	Outcome not evaluated.	Outcome not evaluated.	Outcome not evaluated.	6 (Present).
Gokler et al.^47^ Turkey n = 3,918 14–18 years F 52.2%	Have breakfast (eat breakfast every school day). Not having breakfast^a^.	Age, sex, household income level, and housing	37.6%	Skipping breakfast was associated with obesity in adolescents living in urban areas (OR = 1.33, 95%CI = 1.04; 1.69), but not in those living in rural areas (OR = 1.21, 95%CI = 0.75; 1.93).	Outcome not evaluated.	Outcome not evaluated.	Outcome not evaluated.	7 (Present).
Talat and Shahat^48^ Sharkia Province n = 900 12–15 years F 52.0%	Breakfast consumption frequency: Never^a^ Sometimes Often	Not adjusted.	27.6%	Skipping breakfast was associated with obesity (OR = 3.36, 95%CI = 2.1; 17.6).	Outcome not evaluated.	Outcome not evaluated.	Outcome not evaluated.	7 (Present).
Cayres et al.^49^ Brazil n = 120 11–14 years M 51.7%	Breakfast frequency ≤ 6 days/week.	Sex, Age, Ethnicity, and Abdominal Fat.	47.5% of adolescents reported omitting breakfast less than 1 day/week.	Adolescents who skipping breakfast at least 1 day/week had higher abdominal fat values compared to those who never skip out (35.5% vs. 29.1%, p = 0.002).	There was no association between skipping and CT (p = 0.740), HDL (p = 0.723), LDL (p = 0.862) and TG (p = 0.694).	Outcome not evaluated.	Skipping breakfast was associated with increased SBP (p = 0.040).	8 (Low).
Kim et al.^12^ Korea n = 2,091 10–18 years M 52.8%	Do not eat breakfast more than 5 times a week.	Age, BMI, Daily Energy Consumption, and Energy Percentage.	42.1% and 19.8% of girls and 37.0% and 17.8% of boys skipping breakfast in 1998 and 2010, respectively.	Outcome not evaluated.	Skipping breakfast was associated with hypertriglyceridemia in girls (OR = 2.27, 95%CI = 1.02; 5.31) and increased risk of having high LDL-cholesterol in boys (OR = 5.77, 95%CI = 1.02; 33.28).	Outcome not evaluated.	Outcome not evaluated.	8 (Low).
Morales and Montilva^50^ Venezuela n = 800 15–19 years M 50.6%	Skipping Frequency: Never Rarely or less than once / week, 1–3 times / week 5, 6 and 7 times / week^a^.	NSE	Not shown.	Skipping breakfast was not associated with overall obesity (PR = 0.66, 95%CI = 0.29; 1.47) or central obesity (PR = 0.92, 95%CI = 0.70; 1.22).	Outcome not evaluated.	Outcome not evaluated.	Outcome not evaluated.	9 (Low).
Badr et al.^51^ Asia Occidental n = 2,672 12–16 years M 50.3%	“How often do you have breakfast?” Never^a^ Sometimes Ever	Education, gender, food consumption (fruits, vegetables, milk, fast food and soda), physical activity ≥ 60 min, and time sitting at home..	25.4%	Skipping breakfast was associated with obesity (OR = 1.55, 95%CI = 1.23; 1.95) and overweight (OR = 1.44, 95%CI = 1.16; 1.79).	Outcome not evaluated.	Outcome not evaluated.	Outcome not evaluated.	7 (Present).
Frayon et al.^52^ New Caledonia n = 621 11–16 years F 54.4%	“Do you have breakfast before you go to school?” Skipping No skipping	NSE, age, gender, ethnicity, weight status.	15.3%	Skipping breakfast was associated with overweight for boys (OR = 2,981, 95%CI = 1,460; 6,085), but not among girls (OR = 1,085, 95%CI = 0,537; 2,190). The same trend was found for obesity (boys: OR = 3.301, 95%CI = 1.388; 7.851; girls: OR = 2.291, 95%CI = 0.928; 5.656).	Outcome not evaluated.	Outcome not evaluated.	Outcome not evaluated.	8 (Low).
Zalewska et al.^53^ Poland n = 1,999 18 years F 65.7%	Breakfast Habit: Skipped, < 8 AM, ≥ 8 AM.	Not adjusted.	25.0%	There was no significant difference in breakfast skip prevalence between normal weight and overweight and obese students (p > 0.05).	Outcome not evaluated.	Outcome not evaluated.	Outcome not evaluated.	7 (Present).
De Cnop et al.^54^ Brazil n = 1,749 10–19 years F 50.1%	Breakfast consumption frequency: Regular (daily consumption) Irregular (≤ 6 times/week)^a^	Sex and age.	Not shown.	Skipping breakfast was associated with being overweight in public (OR = 1.48, 95%CI = 1.07; 2.06) and private (OR = 1.57, 95%CI = 1.19) schoolchildren. 2.07); elevated WHR (OR = 1.50, 95%CI = 1.03; 2.19) and high fat% (OR = 1.47, 95%CI = 1.12; 1.95) only in school students private	Outcome not evaluated.	Outcome not evaluated.	Outcome not evaluated.	8 (Low).
Khan et al.^55^ Bangladesh n = 793 12–17 years M 50.0%	5 to 7 days/week (regular breakfast) 0 to 4 days/week (skipping breakfast).	Sex, age, walk to school, involvement in school sports, and family income.	11% skipping every day and 23% consumed ≤ 4 days a week.	Skipping breakfast was associated with overweight (OR = 1.77, 95% CI = 1.11; 2.83) and obesity (OR = 2.62, 95%CI = 1.35; 5.08) after adjustment.	Outcome not evaluated.	Outcome not evaluated.	Outcome not evaluated.	7 (Present).
Silva et al.^56^ Brazil n = 493 10–14 years F 52.3%	Breakfast was considered the first meal of the day, eaten between 5:00 and 9:00 am. Have breakfast Missing Breakfast^a^.	Physical activity, energy consumption and sex.	30.0%	There was no significant association between breakfast skipping and BMI Z-score (p = 0.666), WC (p = 0.640) and% body fat (p = 0.777).	There was no significant association between o breakfast skipping and TC (p = 0.650), HDL (p = 0.766), LDL (p = 0.714) and TG (p = 0.409).	There was no significant association between skipping breakfast and glucose (p = 0.427).	There was no significant association between skipping breakfast and SBP (p = 0.409) and DBP (p = 0.806).	8 (Low).
Tee et al.^57^ Malaysia n = 3,000 13–17 years F 51.8%	Defined as the first time of drinking after a night until 10am on weekdays or until 11am on weekends. Namely breakfast skippers (ate breakfast 0–2 days/week), Irregular breakfast eaters (ate breakfast 3–4 days/week), Regular breakfast eaters (ate breakfast 5–7 days/week).	Age, sex (except for gender analysis), ethnicity, father’s education level, monthly household income and physical activity scores.	15.9%	Girls who skipped breakfast were 38% more likely (95% CI = 1.01; 1.9, p = 0.044) to be overweight or obese than those who ate breakfast regularly. There was no significant association for boys (OR = 1.31, 95%CI = 0.92; 1.87, p = 0.135).	Outcome not evaluated.	Outcome not evaluated.	Outcome not evaluated.	9 (Low).
Werneck et al.^58^ Brazil n = 280 11–18 years M 70.7%	Report of the number of days breakfast was consumed, considering a normal week. Breakfast consumption < 7 days/week^a^.	Sex, chronological age, ethnicity, somatic maturation, and body adiposity (for HOMA-IR analysis).	45.2% of boys and 35.4% of girls skipping breakfast.	Adolescents skipping breakfast had higher body fat (p = 0.002).	Outcome not evaluated.	There was no significant association between skipping breakfast and HOMA-IR (p = 0.432)	Outcome not evaluated.	8 (Low).
Forkert et al.^59^ Europe and Brazil HELENA n = 2,371 12,5–17,5 years F 54.8% BRACAH n = 991 14–18 years F 54.5%	HELENA Agree with the statement: “I often skip breakfast” classified into 7 categories ranging from strongly disagree (1) to strongly agree (7) with category 4 being placed as neither agreeing nor disagreeing by categorizing the variables into: I don’t skip breakfast (1 to 3) Skip Breakfast (5–7) BRACAH Does the meal at home or not, being categorized as: Don’t skip breakfast Skip the breakfast.	Age, maternal education level, cities participating in the HELENA-CSS study only.	HELENA 44.5% of girls and 35.9% of boys skipped breakfast. BRACAH 37.8% of girls and 34.6% of boys skipped breakfast.	HELENA Skipping breakfast was associated with overweight and abdominal obesity in adolescents of both sexes (Female: Overweight p < 0.001, WC p = 0.025, WHR p = 0.001; Male: Overweight p < 0.001, CC p < 0.001, RCE p < 0.001). BRACAH Skipping breakfast was associated with overweight (p = 0.004) and abdominal obesity (WC: p = 0.038, WHR: p = 0.005) in male adolescents only.	Outcome not evaluated.	Outcome not evaluated.	Outcome not evaluated.	8 (Low).
Mustafa et al.^60^ Malaysia n = 795 13 years F 63.0%	Number of days any food or drink was reported for breakfast. Categorized in: daily 4 to 6 days/week 1 to 3 days/week 0 days/week^a^.	Physical activity, sex, ethnicity, smoking and alcohol consumption.	10% of adolescents skipped breakfast.	The adolescents who skipped breakfast had higher BMI compared to those who consumed daily (19.9 vs. 19.2 Kg/m^2^, p = 0.003).	Compared with daily breakfast consumers, adolescents who never had breakfast had higher serum TC (4.6 vs. 4.8 mmol / L, p = 0.01) and LDL (2.7 vs. 2.9 mmol / L, p = 0.01).	There was no significant association between skipping breakfast and blood glucose (p = 0.79).	There was no significant association between skipping breakfast and SBP (p = 0.32) and DBP (p = 0.45).	7 (Present).

F: female; M: male; EDE-Q: Examination Questionnaire for Eating Disorder; BF: body Fat; BMI: body mass index; CI: confidence Interval; DBP: diastolic blood pressure; HbA1c: glycosylated hemoglobin; HDL: high density lipoprotein; HOMA-IR: homeostatic model insulin resistance; LDL: low density lipoprotein; NSE: socioeconomic level; OR: odds ratio; PR: prevalence ratio; SBP: systolic blood pressure; SD: standard deviation; REC: ready to eat cereals; RR: relative risk; TC: total cholesterol; TG: triglycerides; WC: waist circumference; WHR: waist-height ratio.

^a^ Skipping breakfast.

^1^ Viswanathan e Berkman^19^; ^2^ The proportion of females and males was not reported; ^3^ Study follow-up period; ^4^ Only 2 participants of 20 years and were classified with normal weight.

### Inclusion and Exclusion Criteria of the Studies

This review included cross-sectional and longitudinal observational studies published between 2008 and 2019 in English, Spanish, or Portuguese, conducted with adolescents (10–19 years old, as defined by the World Health Organization)^18^, which have estimated the association of breakfast skipping with at least one of the cardiometabolic risk factors (markers of body adiposity, blood pressure, systemic arterial hypertension, serum lipid, and glucose levels).

The articles including adolescents with mental disorders, kidney disease, HIV, cancer, Down syndrome, and others were excluded from the review; those including other age groups, in addition to individuals aged between 10 and 19 years, and articles that did not present separate results for this age group; non-empirical records, opinions and editorials, case studies, summaries of scientific events that were not published as full papers, intervention studies and animal studies were also excluded.

### Review Process

The articles were selected by two independent researchers and disagreements were discussed and analyzed at subsequent meetings. After the search, the Mendeley reference management tool was used to exclude duplicate records, and an initial screening was performed to reject records whose title and/or abstract information failed the inclusion criteria. If abstracts were unavailable or provided insufficient results, the article was selected for full reading. After analyzing the articles previously selected for full reading, reviewers included studies with results on the association of breakfast skipping with cardiometabolic risk factors (Figure).

**Figure:**
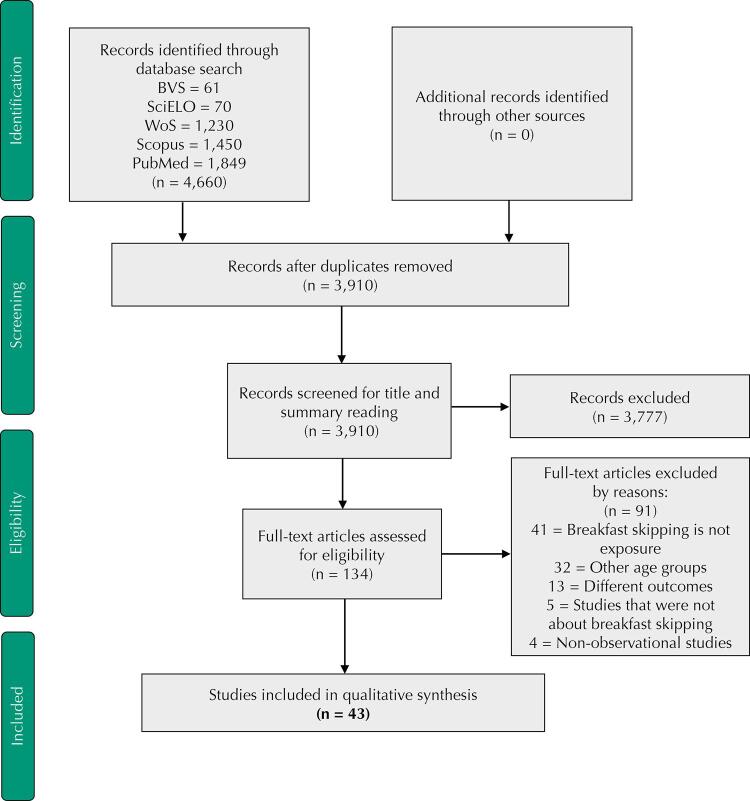
PRISMA flow diagram of study selection process.

The studies were presented according to the following characteristics: (1) identification data with citation of the author(s), year of publication and place of study; (2) number of individuals evaluated, age and sex of the sample studied; (3) study outcome(s); (4) breakfast skipping assessment; (5) control of confounding factors and (6) main results (prevalence of breakfast skipping, association of breakfast skipping with markers of body adiposity, serum lipid levels, glucose metabolism (insulin resistance, fasting glucose and glucose intolerance) and blood pressure). Table 1 shows this information in chronological order.

### Evaluation of Study Quality

The studies included in this systematic review underwent independent quality and risk of bias review by two reviewers. The instrument used was the Research Triangle Institute (RTI) Item Bank on Bias Risk and Accuracy of Observational Studies proposed and validated by Viswanathan & Berkman^19^ to assess risk of bias and accuracy of observational studies.

The RTI Item Bank includes several study designs, and the authors provided instructions on which items to use depending on the studies being evaluated. Considering the authors’ recommendations and the types of study included in this review, ten items were used to evaluate cross-sectional studies (sample definition and selection, consistency of information, outcomes, comparability of analysis, analysis results, interpretation of results, and funding) and thirteen items for cohort studies (three additional questions on follow-up time and impact of losses). Thus, a cross-sectional study with three or more key items classified as negative or unclear or a cohort study with four or more negative or unclear items were classified as at risk of bias (Table 1).

### Evaluation of the Quality of Evidence

This evaluation aims to provide greater reliability to the critical analysis of the findings. In this study, the Grading of Recommendations Assessment, Development and Evaluation (GRADE) System was used for each analyzed outcome^20^. The following criteria were used to evaluate the studies: design (randomized clinical trial starts with high quality and observational study with low); factors responsible for reducing evidence level (methodological limitations, indirect evidence, imprecision, and publication bias) and factors that contribute to raising the evidence (large magnitude of effect, dose-response gradient, and residual confounding factors). After evaluation according to the GRADE rating, the studies were classified into four quality of evidence levels: High (A) – There is strong confidence that the true effect/association is close to that estimated; Moderate (B) – There is moderate confidence in the estimated effect/association; Low (C) – Confidence in the effect/association is limited; and, Very low (D) – Confidence in the effect/association estimate is very limited^20^ (Table 2).

**Table 2:** Relationship between breakfast skipping and cardiometabolic risk factors with classification of the quality of evidence according to the GRADE system.

Exposure	Outcome	Positive association	Negative association	No association	Summary of evidence (Grade)^a^
Breakfast skipping	Glycemic profile	-	-	Alexander et al.^29^ Shafiee et al.^41^ Silva et al.^56^ Werneck et al.^58^ Mustafa et al.^60^	No association (C)
Breakfast skipping	Lipid profile	Shafiee et al.^41^ Kim et al.^12^ Mustafa et al.^60^	-	Cayres et al.^49^ Silva et al.^56^	Positive association (C)
Breakfast skipping	Blood pressure	Kollias et al.^31^ Shafiee et al.^41^ Cayres et al.^49^	-	Silva et al.^56^ Mustafa et al.^60^	Positive association (C)
Breakfast skipping	Markers of body adiposity	Timlin et al.^21^ Wang et al.^16^ Cayres et al.^22^ Wu et al.^11^ Harding et al.^25^ Maddah^26^ Sánchez et al.^28^ Alexander et al.^29^ Croezen et al.^30^ Maddah et al.^32^ Sun et al.^33^ Deshmukh- Taskar et al.^34^ Kapantais et al.^35^ Maddah and Nikooyeh^36^ Thompson-McCormick et al.^37^ Kuriyan et al.^39^ Vaezghasemi et al.^40^ Shafiee et al.^41^ Boričic et al.^42^ Díez-Navarro et al.^43^ Faizi et al.^44^ Garcia-Continente et al.^45^ Gokler et al.^47^ Talat and Shahat^48^ Cayres et al.^49^ Badr et al.^51^ Frayon et al.^52^ De Cnop et al.^54^ Khan et al.^55^ Tee et al.^57^ Werneck et al.^58^ Forkert et al.^59^ Mustafa et al.^60^	-	Hassan et al.^23^ Hassan et al.^24^ Mota et al.^27^ Kim and So^38^ Garg et al.^46^ Morales and Montilva^50^ Zalewska et al.^53^ Silva et al.^56^	Positive association (C)

^a^ Quality of evidence (GRADE system): A – High; B – Moderate; C – Low; D – Very Low.

## RESULTS

In the databases searched, 4,660 records were identified and 3,910 remained after deletion of duplicates. Then, the titles and abstracts were read, and 134 articles were selected for full reading, of which 91 did not meet the inclusion criteria; therefore, a total of 43 articles were included in this systematic review (Figure).

Table 1 shows the summary of the main characteristics of the 43 studies involving 192,262 participants from 10 to 19 years old. Six studies are longitudinal (cohort)^11,16,21^ and thirty-seven are cross-sectional studies^12,24^. In twenty-two articles, the population was predominantly female^16,21,25,26,29,31,32,34^ and four of them studied only adolescent girls^25,31,35,36^. Three articles did not describe the frequency of each sex^21,24,41^.

The selected studies were conducted in 22 countries. Seventeen studies were conducted in Asian countries^11,12,26,32,33,36,38,39,41,44,46^, twelve in European countries^25,27,28,30,31,35,40,42,43,45,53,59^, twelve in countries of the American continent^16,21^ and two in Oceanian countries^37,52^. The countries with the highest number of studies were Brazil^22^, United States^16,21,29,34^, and Iran^26,32,36,41^(Table 1).

Three studies evaluated markers of body adiposity, blood pressure, and glycemic and lipid profile^41,56,60^, one evaluated markers of body adiposity, lipid profile, and blood pressure^49^; two assessed markers of body adiposity and glycemic profile^29,58^; one article evaluated only lipid profile^12^; one only blood pressure^31^ and thirty-five articles evaluated only markers of body adiposity (body mass index (BMI), waist circumference (WC), body fat %, and waist-height ratio (WHR))^11,16,21^.

The definition of breakfast skipping presented many variations; twenty-three articles assessed skipping by frequency of breakfast consumption; twelve studies considered the number of days per week (0 days/week^60^, 0–2 days/week^35,57,41^, 0–3 times/week^16,40^, 0–4 days/week^12,55^, 0–5 days/week^22^, < 2 times/week^44^, ≤ 6 times/week^49,58^); one study considered breakfast skipping when frequency of consumption was reported as rarely^33^; two considered skipping as never or almost never^23,24^; and eight studies considered skipping as never before^21,26,32,38,42,45,48,51^. Four articles evaluated skipping by the frequency of breakfast skipping (sometimes or always vs. rarely^25^, 0 to 7 days^30,37^ and 5, 6, and 7 times/week^50^). Seven articles used the “skip yes/no” option^11,27,39,46,52,53,59^ and nine defined skipping according to breakfast consumption: yes/no^28,31^, regular/non-regular^36,54^, presence/absence^43,47,56^ and whether study participants did not report food or drink consumption in the R24h^29,34^ (Table 1).

The prevalence of breakfast skipping ranged from 0.7%^33^ to 94%^27^, according to the different definitions of breakfast skipping used in the articles. Twelve studies did not report the prevalence of skipping breakfast^11,22,25,26,30,32,36,39,40,42,50,54^. Twenty-six^12,16,22^ (60.5%) studies were classified with low risk of bias (Table 1).

The six longitudinal studies (cohort) evaluated only the markers of body adiposity^11,16,21^. Four studies found significant association between breakfast skipping and markers of body adiposity^11,16,21,22^, that is, adolescents who skipped breakfast showed an increase in BMI, and body and abdominal fat over time. The analyzes were adjusted for potential confounding factors related to sociodemographic, economic, lifestyle and sexual maturation characteristics (Table 1).

In relation to cross-sectional studies, breakfast skipping was associated with higher prevalence of body adiposity. Twenty-nine articles (78.4%) found a significant association between breakfast skipping and excess adiposity^25,26,28^. However, Alexander et al.^29^ found association only for Intra-Abdominal Adipose Tissue, Gokler et al.^47^ found association only in adolescents living in urban areas, Garcia-Continente et al.^45^, Tee et al.^57^ found association only for female adolescents and Frayon et al.^52^ found association only for males.

Of these thirty articles that found breakfast skipping associated with excess adiposity, twenty-one performed adjusted analysis for potential confounders, such as: age, sex, race/ethnicity, socioeconomic status, total energy consumption, pubertal stage and physical activity^25,26,29,30,33,34,36,37,41,42,45,47,49,51,52,54,55,57^. On the other hand, five studies found no association between breakfast skipping and body adiposity^38,46,50,53,56^, of which two made no adjustments for potential confounding factors^46,53^. In summary a significant positive association between the breakfast skipping and excess adiposity is evidenced with a GRADE C classification (Table 2).

Among the articles included in this review, eight evaluated the association of breakfast skipping with cardiometabolic risk factors (glucose metabolism, blood pressure, and serum lipid levels), all of which are cross-sectional^12,29,31,41,49,56,58,60^.

Of these studies, five investigated the association between breakfast skipping and lipid profile^12,41,49,56,60^, and three found a statistically significant association; therefore, adolescents who omitted breakfast had higher levels of total cholesterol (TC), low density lipoprotein (LDL) and triglyceride (TG)^12,41,60^. Five of the eight articles investigated the association between breakfast skipping and glycemic profile (fasting glucose, Homeostatic Insulin Resistance Assessment Model (HOMA-IR), acute insulin response, and insulin sensitivity), but none of them found a significant association^29,41,56,58,60^.

Regarding blood pressure, five articles evaluated the association between breakfast skipping and high blood pressure^31,41,49,56,60^; and three found a significant association, i.e., adolescents who skipped breakfast had higher levels of systolic blood pressure (SBP)^31,41,49^.

In summary a significant positive association for lipid profile and blood pressure was found, whereas for glycemic profile there was no association, all risk factors were rated GRADE C.

All studies investigating the association of breakfast skipping with cardiometabolic risk factors considered the potential confounders: age^12,29,31,41,49,58^, sex^29,41,49,56,58,60^, ethnicity^49,58,60^, socioeconomic status^41^, BMI^12,31,41^, pubertal stage^29,58^, energy intake^12,29,56^, abdominal adiposity^49^, total adiposity^29,58^, physical activity^56,60^, and smoking and alcoholic beverage^60^.

## DISCUSSION

This systematic review included 43 studies that evaluated the association between breakfast skipping and cardiometabolic risk factors in adolescents. The prevalence of breakfast skipping varied widely between studies. Adolescents who skipped breakfast were more likely to have cardiometabolic risk factors, e.g., excess adiposity, higher levels of total cholesterol, low density lipoprotein, triglycerides, and blood pressure. However, the association between breakfast skipping and glycemic profile is unclear, since only five studies were included, and none found a significant association. The impact of the content of macronutrients present at breakfast^29^ and adjustment for body adiposity^29,41,58,60^, and quality of diet^60^ can explain those associations.

Of the studies, 78.4% found that breakfast skipping is associated with excess body fat in adolescents. Intiful and Lartey^61^ attribute this association to adolescents who omit breakfast being more likely to eat other meals, such as lunch and dinner, irregularly and to eat unhealthy food between meals, such as non-nutritious snacks and fast foods. Thus, adolescents who skip breakfast consume more fat and less fiber, vitamins, and minerals^4^ and are less physically active, leading to an imbalance between energy consumption and expenditure^62^.

Wang et al.^16^ and Cayres et al.^22^ showed that breakfast skipping is associated with weight gain, since adolescents who skipped this meal spent a longer period fasting (night and morning), with increased ghrelin release^63^, consequently, increased appetite, increasing the probability of hyperphagia and body fat accumulation^64^.

A potential confounding factor in assessing the association between breakfast skipping and body adiposity is energy intake, since individuals who skip breakfast tend to consume high energy density foods, or they may consume more energy from other meals throughout the day^16,49^. Four studies in this review evaluated the association of breakfast skipping with body adiposity, adjusting for total energy consumption; in three of them, the association remained even after adjustment^21,29,34^.

Three articles found an association between breakfast skipping and abnormal lipid profile^12,41,60^ in addition to the observed propensity for excess body adiposity. The main factors that may explain this association are related to increased appetite and high energy intake after fasting^12^. Breakfast skipping also induces insulin resistance and may play an important role in the development of dyslipidemia^65^. Hyperinsulinemia may increase apolipoprotein B-48 secretion by stimulating chylomicrons formation in the small intestine and inducing postprandial hyperlipidemia. Next, lipoprotein lipase, which hydrolyzes the central chylomicrons triglycerides^66^, is inhibited, leading to increased hepatic lipase activity, which hydrolyzes LDL-cholesterol and HDL-cholesterol, triggering the decrease in HDL-cholesterol and the increase of small and dense LDL-cholesterol arterial particles^67^.

Regarding blood pressure increase in adolescents who omit breakfast, three articles in this review found a significant association^31,41,49^. The mechanisms that explain this association are still unclear, but they seem to be related to body adiposity, which may be the result of skipping breakfast, as mentioned above. Vanderlei et al.^68^ argue that increased body fat is related to higher sympathetic activity, because adiposity is associated with increased oxidative stress, increasing stimulation in specific brain regions that control sympathetic activity^69^. However, the associations found by Kollias et al.^31^, Shafiee et al.^41^, and Cayres et al.^49^ remained significant for SBP, even after adjustment for indicators of body adiposity.

The five articles that evaluated breakfast skipping and glucose metabolism-related variables found no significant association^29,41,56,58,60^. Alexander et al.^29^ reported that this association was not supposed to be found by the impact of the macronutrient content in breakfast on metabolic parameters, but the study did not evaluate the type of breakfast, therefore, its influence in the results cannot be stated. According to Tolfrey and Zakrzewski^70^, breakfast with high-fiber, low-glycemic cereals is associated with increased glycemic control and satiety.

The metabolic effects of breakfast can also be explained by their impact on the circadian cycle, a period in which the environment influences the transcription of some genes, mainly by light variations and by the time of feeding, which helps synchronize this system^71^. When food is restricted during a period, such as breakfast skipping, i.e., the fasting period after the sleeping period increases, circadian cycles may be out of sync, causing changes in certain genes that regulate lipid and glucose metabolism^1^.

The variation in the results found in the articles may have been influenced by the different ways of collecting information on breakfast consumption and the definition of skipping breakfast. The definition most used by the studies considered the frequency of breakfast consumption (“rarely”, “never” and number of days/week).

Dialektakou et al.^72^ stressed that breakfast skipping is associated with BMI, but this association depends on how skipping is defined. Among Greek adolescents, the authors found that all variables corresponding to breakfast consumption on the day of data collection were associated with both BMI and overweight or obesity. However, few associations remained significant when considering breakfast consumption in the previous week; which may have been because adolescents’ recollections of omitting breakfast in the previous week were less accurate than information reported on the day of data collection.

Among Brazilian adolescents, Hassan et al.^23^ also found that the results depend on how breakfast skipping defined and on how the variable was categorized; they noted that the prevalence of breakfast skipping ranged from 3.6 % when the baseline was last week to 39.0% when we asked the teenagers if they had eaten any solid food in the morning of the interview.

In this systematic review, we observed that, in general, studies that considered breakfast skipping 5 or more times or every day showed a higher frequency of positive association between skipping this meal and the respective outcomes: lipid profile^12,40,59^, blood pressure^40,48^ and body adiposity markers^11,12,21,22,26,30,32,35,37^. Thus, a cutoff point of breakfast skipping almost every and every day possibly allows a better assessment of the association between this behavior and cardiometabolic risk factors, because adolescents who report breakfast skipping every day probably have this habit over months and years, resulting in metabolic and body composition changes.

In the cohort study conducted by Timlin et al.^21^ with a 5-year follow-up, they considered a cutoff point of breakfast skipping every day and observed that adolescents who omitted breakfast had a greater increase in the mean BMI. Cayres et al.^22^ observed similar results, which also considered the cutoff point of skipping in 5 days, follow-up was 12 months and abdominal fat and total body fat of the adolescents who skipped breakfast increased more than those who ate regularly.

Thus, the definition and standardization of the category defining breakfast skipping may influence the results and their interpretation. This systematic review found a wide variation in the definitions of breakfast skipping, hindering the comparison between the results. According to Hassan et al.^23^, a standard definition of breakfast skipping in the scientific literature is essential to improve comparisons among studies and results interpretation.

The American Heart Association recently proposed definitions to improve and standardize the assessment of consumption and skipping of meals^1^: breakfast as the first meal of the day that breaks the fast after the longest period of sleep, eaten within 2 to 3 hours of waking, consisting of food or drink from at least one food group and consumed in any location. Other meals were defined as consumption occasions that provide ≥ 15% of the total energy intake and snacks as consumption occasions that provide < 15% of the total energy intake^1^.

In addition, Food Guide for the Brazilian population^73^ recommends the consumption of fresh or minimally processed foods for breakfast, such as milk, coffee, eggs, fruits, and preparations based on cereals or tubers, since the nutritional composition of this meal is important and the consumption of certain foods for breakfast can have a protective effect against cardiometabolic risk factors, such as BMI, total cholesterol, and LDL^60^.

Standardizing the definition of breakfast skipping is necessary to enable the aggregation of results and the summarization of effects, and to better evaluation and understanding of scientific evidence in the association between breakfast skipping and cardiometabolic risk factors in adolescents. This can subsidize public policies and technical advice directed to schools aiming at instructing on the effects and risks of skipping breakfast on the cardiometabolic health of adolescents and promoting the daily consumption of this meal.

The studies included in this systematic review showed a significant association with excess adiposity, lipid profile, and blood pressure. In summary, the positive association remained for these outcomes with quality, according to the GRADE rating, C – low. As for the glycidic profile, no association was observed, and the quality of evidence was also C – low. Despite the low quality of the evidence of this study’s findings, we observed that they agree with what other studies with adolescents verified, thus, breakfast skipping was associated with cardiometabolic risk factors^5,10,15^. The low quality of the evidence from the studies was also mainly due to the observational design and inconsistency.

The conflicts of interest towards the financing of studies by the food industry is also important to point out. Some studies^16,41,44^ were financed by the food industries that manufacture breakfast cereals and despite the researchers reporting no conflict of interest, the food industry has an interest in promoting the habit of having breakfast, because the consumption of breakfast cereals generally occurs in this meal. Breakfast cereals are also classified as ultra-processed food products, that is, they contain high levels of added sugars, salt, and unhealthy fats, thus being unhealthy. Pagliai et al.^74^ systematic review and meta-analysis, which analyzed ultra-processed and chronic diseases, showed a relation between the consumption of ultra-processed products and increased risk of obesity, metabolic syndrome, and cardiovascular diseases.

The strengths of this review include the search in various databases, the selection and evaluation of articles by peers, the evaluation of the quality of the included studies and the focus only on adolescents aged 10 to 19 years old. Among the limitations is the inclusion of longitudinal studies that evaluated only the markers of body adiposity and the impossibility of performing statistical synthesis through meta-analysis, due to the different definitions of breakfast skipping and statistical analysis methodologies adopted by the studies.

## CONCLUSION

The results suggest that breakfast skipping is associated with cardiometabolic risk factors in adolescents aged 10 to 19 years. However, considering the low quality of the evidence, they should be interpreted carefully. We emphasize the importance of standardizing the breakfast skipping definition for better use as an exposure factor in assessing cardiometabolic risk factors and we recommend carrying out studies with longitudinal designs to raise the level of evidence and ensure temporality and consistency of results in different confusion scenarios.
